# Supplementary material for: Heat adaptation of phage T7 under an extended genetic code
Source: Virus Evol. 2021 Dec 1;7(2):veab100. doi: 10.1093/ve/veab100 (PMC8923235; doi:10.1093/ve/veab100)
Supplement: veab100_Supp [file veab100_supp.zip › Sequencing_Analysis.zip!/Sequencing_Analysis/A1/evidence/JC_SIDE_2_60.html]

BRESEQ :: Evidence


| New junction evidence | | | | | | | | | | | |
| --- | --- | --- | --- | --- | --- | --- | --- | --- | --- | --- | --- |
|  | | seq id | position | reads (cov) | reads (cov) | score | skew | freq | annotation | gene | product |
| \* | ? | NC\_001604.1 | = 13568 | 84 (0.030) | 12 (0.000) | 7/586 | 43.0 | 8.7% | intergenic (–/–) | *–/–* | –/– |
|  | ? | NC\_001604.1 | 14339 = | 169 (0.060) |  |  |  |  | intergenic (–/–) | *–/–* | –/– |
| Rejected: Coverage evenness skew score above cutoff. | | | | | | | | | | | |
| Rejected: Frequency below/above cutoff threshold. | | | | | | | | | | | |

Only 100 of 813 total aligned reads displayed.  

`AGGCTGGACTTGGACAAGACAGACTGGCTGGAAGAAACCAGAAGTTACCAAGCTGAGTCATAAGGTGTTCGATAAGGACACTATGACCCACATCAAGGCTGGTGATTGGGTTAAGGTTGACATGGGAGTTGTTGGTGGATACGGCTACGTCCGCTCAGTTAGTGGCAAATATGCACAAGTGTCATACATCACAGGTGTTACTCCACGCGGTGCAATCGTTGCCGATAAGACCAACATGATTCACACAGGTTTCTTGACAGTTGTTTCATATGAAGAGATTGTTAAGTCACGATAATC‑AATAGGAGAAATCAATATGATCGTTTCTGACATCGAAGCTAACGCCCTCTTAGAGAGCGTCACTAAGTTCCACTGCGGGGTTATCTACGACTACTCCACCGCTGAGTACGTAAGCTACCGTCCGAGTGACTTCGGTGCGTATCTGGATGCGCTGGAAGCCGAGGTTGCACGAGGCGGTCTTATTGTGTTCCACAACGGTCACAAGTATGACGTTCCTGCATTGACCAAACTGGCAAAGTTGCAATTGAACCGAGAGTTCCACCTTCCTCGTGAGAACTGTATTGACACCCTTGTGTTGTCAC  >  NC_001604.1/14040‑14638`  

                                                                                                                                                                                                                                                                                                            `|`                                                                                                                                                                                                                                                                                                             
`gagtcatactcagagtcaacagacTGGCTGGAAGAAACCAGAAGTTACCAAGCTGAGTCATAAGGTGTTCGATAAGGACACTATGACCCACATCAAGGCTGGTGATTGGGTTAAGGTTGACATGGAAGTTGTTGGTGGATACGGCTACGTCCGCTCAGTTAGTGGCAAATATGCACAAGTGTCATACATCACAGGTGTTACTCCACGCGGTGCAATCGTTGCCGATAAGACCAACATGATTCACACAGGTTTCTTGACAGTTGTTTCATATGAAGAGATTGTTAAGTCACGATAATC‑ACTa                                                                                                                                                                                                                                                                                                            >  1:435750/19‑301 (MQ=255)`  
 `acgtggttctggcgcactacgccaactatctgatactattattgcccttgagcgtaatcagcaaggcgatatgcctaaccttgtcctcgttcgtattctcaagtgccgctttactggtgatactggtatcgctggctacatggaatacaacaaggaaaccggatggcttgaaccatcaagttactcaggggaagaagagtcacactcagagtcaacagactggtccaacgacactgacttctgacaggaatcttgaCAGTTGTTTCATATGAAGAGATTGTTAAGTCACGATAATC‑AATAg                                                                                                                                                                                                                                                                                                           >  1:482213‑M2/257‑301 (MQ=255)`  
 `acgtggttctggcgaactacgcaaactatcggatactattagtgcccttgagcgtaatcagcaaggcgatatgcctaaccttgtcctcgttcgtattctcaagtgccgctttactggtgatactggtatcgctggctacatggaatacaacaaggaaaccggatggcttgaaccatcaagttactcaggggaagaagagtcacactcagagtcaacagactggtccaacgacactgacttctgacaggattcttgaCAGTTGTTTCATATGAAGAGATTGTTAAGTCACGATAATC‑AATAg                                                                                                                                                                                                                                                                                                           <  1:152187‑M2/45‑1 (MQ=255)`  
    `gggttctggcgaactacgccaactatctgatacgattattgcccttgagcgtaatcagcaaggcgatatgcctaaccttgtccgcgttcgtattctcaagtgccgctttactggtgatactggtatcgctggctacatggaatacaacaaggaaaccggatggcttgaaccatcaagttactcaggggaagaagagtcacactcatagtcaacagactggtccaacgacactgacttctgacaggattcttgaCAGTTGTTTCATATGAAGAGATTGTTAAGTCACGATAATC‑AATAGgag                                                                                                                                                                                                                                                                                                        <  1:459629‑M2/48‑1 (MQ=255)`  
      `gttctggcgcactacgccaactatctgatactattattgcccttgagcgtaatcagcaaggcgatatgcctcaccttgtcctcgttcgtattctcaagtgccgctttactggtgttactggtatcgctggatacatggaatacaacacggaaactggatggcttgaaccatctagttacttagggtaagaagagtcacactcagagtcaacagactggtccaacgacactgacttctgacatgattcttgaCAGTTGTTTCATATGAAGAGATTGTTAAGTCCCGACACTC‑AATAGGAGaa                                                                                                                                                                                                                                                                                                      >  1:356058‑M2/252‑301 (MQ=255)`  
              `gacagacgccaactatctgatactattattgcccttgagcgtaatcagcaaggcgatatgcctaaccttgtcctcgttcgtattctcaagtgccgctttactggtgatactggtatcgctggctacatggaatacaacaaggaaaccggatggcttgaaccatcaagttactcaggggaagaagagtcacactcagagtcaacagactggtccaacgacactgacttctgacaggattcttgaCAGTTGTTTCATATGAAGAGATTGTTAAGTCACGATAATC‑AATAGGAGAAATCAatat                                                                                                                                                                                                                                                                                              <  1:25112‑M2/58‑1 (MQ=255)`  
                 `ctacgccaactatctgatactattattgcccttgagcgtaatcagcaaggcgatatgcctaaccttgtcctcgttcgtattctcaagtgccgctttactggtgatactggtatcgctggctacatggaatacaacaaggaaaccggatggcttgaaccatcaagttactcaggggaagaagggtcacactcagagtcaacagactggtccaacgacacttacttctgacaggtttcttgaCAGTTGTTTCATATGAAGAGATTGTTAAGTCAcgataata‑aataggagctgtatcttatac                                                                                                                                                                                                                                                                                           >  1:340687‑M2/241‑288 (MQ=255)`  
                   `acgcaaactatctgaaactatcattgtcctttagcgtaatcagcaaggcgatatgcctacccttgtcctcgttcgtattctcaagtgacgctttactggtgatactggtatcgctggctacatggaatacaacaatgaaaacggatggcttgaaccatcaagttactcaggggaagaagagtcacactcagagtcaaaatactggtccaacgaccctgacttctgacaggattcttgaCAGTTGTTTCATATGAAGAGATTGTTAAGTCACGATAATC‑AATAGGAGAAATCAATATGATCg                                                                                                                                                                                                                                                                                         <  1:133596‑M2/63‑1 (MQ=255)`  
                          `ctatctgatactattattgcccttgagcgtaatcagcaaggcgatatgcctaaccttgtcctcgttcgtattctcacgtgccgctttactggtgatactggtatcgctggctacatggaatacaacaaggaaaccggatggcttgaaccatcaagttactcaggggaagaagagtcacactcagagtcaacagactggtccaacgacactgacttctgacaggattcttgaCAGTTGTTTCATATGAAGAGATTGTTAAGTCACGATAATC‑AATAGGAGAAATCAATATGATCGTTTCTGa                                                                                                                                                                                                                                                                                  >  1:500158‑M2/232‑301 (MQ=255)`  
                          `ctatctgatactattattgcccttgagcgtaatcagcaaggcgatatgcctaaccttgtcctcgttcgtattctcaagtgccgctttactggtgatactggtatcgctggctacatggaatacaacaaggaaaccggatggcttgaaccatcaagttactcaggggacgaagagtaacaatcagagtcaacagactggtacaacgacactgccttctgacaggattcttgaCAGTTGTTTCATATGAAGATATTGTTAAGTCACGATAATC‑AATAGGAGAAATCAATATGATCGTGTCTGa                                                                                                                                                                                                                                                                                  >  1:142079‑M2/232‑301 (MQ=255)`  
                           `tatctgatactattattgcccttgagcgtaatcagcaaggcgatatgcctaaccttgtcctcgttcgtattctcaagtggcgctttactggtgatactggtatcgctggctacatggaagacaaaaaggaaacaggatggcttgaaccatcaagttactcaggggaagaagagtcacactcagagtcaacagactggtccaacgacactgacttctgacaggattcttgaCAGTTGTTTCATATGAAGAGATTGTTAAGTCACGATAATC‑AATAGGAGAAATCAATATGATCGTTTCTGAc                                                                                                                                                                                                                                                                                 <  1:309157‑M2/71‑1 (MQ=255)`  
                            `atctgatactattattgcccttgagcgtaatcagcaaggcgatatgcctaaccttgtcctcgttcgtattctcaagtgccgctttactggtgatactggtatcgctggctacattgaatacaacaaggaaaccggatggcttgaaccatcaagttactcaggggaagaagagtcacactcagagtcaacagactggtccaacgacactgacttctgacaggattcttgaCAGTTGTTTCATATGAAGAGATTGTTAAGTCACGATAATC‑AATAGGAGAAACAATTATGATCGTTTCTGACa                                                                                                                                                                                                                                                                                >  1:106456‑M2/230‑301 (MQ=255)`  
                                       `ttattgcccttgagcgtaatcagcaaggcgatatgcctaaccttgtcctcgttcgtattctcaagttccgctttactggtgatactggtatcgctggctacatggaatacaacaaggaaaccggatggcttgaaccatcaagttactcaggggaagaagagtcaccctcagagtcaacagactggtccaacgacactgacttctgacaggattcttgaCAGTTGTTTCATATGAAGAGATTGTTAAGTCACGATAATC‑AATAGGAGAAATCAATATGATCGTTTTTGAAATCGAAGCTAAc                                                                                                                                                                                                                                                                     >  1:157988‑M2/219‑301 (MQ=255)`  
                                           `tgcccttgagcgtaatcagcaaggcgatatgcctaaccttgtgcgcgttcgtattctcaagggccgatttaatggtgatactggtatcgctggctacatagaatacaacaaggaaaccggatggcttgaaacataaagttactcaggggaagaagagtaacactcagagtcaacagactgggccaaagacactgacttctgacaggattcttgaCAGTTGTTTCATATGAAGAGATTGTTAAGTCACGAGAATC‑AATAGGAGAAATCAATATGATCGTTTCTGACATCGAAGCTAACGccc                                                                                                                                                                                                                                                                 <  1:47066‑M2/87‑1 (MQ=255)`  
                                           `tgcccttgagcgtaatcagcaaggcgatatgcctaaccttgtcctcgttcgtattctcaagtgccgctttactggtgatactggtatcgctggctacatggaatacaacaaggaaaccggatggcttgaaccatcaagttactcaggggaagaagagtcacactcagagtcaacagactggtccaacgacactgacttctgacaggtttcttgaCAGTTGTTTCATATGAAGAGATTGTTAAGTCACGATAATC‑AATAGGAGAAATCAATATGATCGTTTCTGACATCGAAGCTAACGccc                                                                                                                                                                                                                                                                 <  1:451636‑M2/87‑1 (MQ=255)`  
                                                   `ggcgtaatcagcaaggcgatatgcctaaccttgtcctcgttcgtattctcaagtgccgctttactggtgatactggtatcgctggctacatggaatacaacaaggaaaccggatggcttgaacaatcaagttactcaggggaagaagagtcacactcagagtcaacagactggtccaacgacactgacttctgacaggattcttgaCAGTTGTTTCATATGAAGAGATTGTTAAGTCACGATAATC‑AATAGGAGAAATCAATATGATCGTTTCTGACATCGAAGCTAACGCCCTCTTagag                                                                                                                                                                                                                                                         <  1:1793‑M2/95‑1 (MQ=255)`  
                                                   `agcgtaatcagcaaggcgatatgcctaaccttgtcctcgttcgtattctcaagtgccgctttactggtgatactggtatcgctggctacatggaatacaacaaggaaaccggatggcttgaaccatcaagttactcaggggaagaagagtcacactcagagtcaacagactggtccaacgacactgacttctgacaggattcttgaCAGTTGTTTCATATGAAGAGATTGTAAAGTCACGATAATC‑AATAGGAGAAATCAATATGATCGTTTCTGACATCGAAGCTAACGCCCTCTTagag                                                                                                                                                                                                                                                         <  1:186651‑M2/95‑1 (MQ=255)`  
                                                   `agcgtaatcagcaaggcgatatgcctaaccttgtcctcgttcggattctcaagtgccgctttactggttatactggtatcgctggctacatggaatacaacaaggaaaccggatggcttgaaccatcaagttactcaggggaagaagagtcacactcagagtcaacagactggtccaacgacactgacttctgacaggattcttgaCAGTTGTTTCATATGAAGAGATTGTTAAGTCACGATAATC‑AATAGGAGAAATCAATATGATCGTTTCTGACATCGAAGCTAACGCCCTCTTagag                                                                                                                                                                                                                                                         <  1:427775‑M2/95‑1 (MQ=255)`  
                                                     `cgtaatcagcaaggcgatatgcctaaccttgtcctcgttcgtattctcaagtgccgctttactggtgatactggtatcgctggctacatggaatacaacaaggaaaccggatggcttgaaccatcaagttactcaggggaagaagagtcacactcagagtcaacagactggtccaacgacactgacttctgacaggattcttgaCAGTTGTTTCATATGAAGAGATTGTTAAGTCACGATAATC‑AATAGGAGAAATCAATATGATCGTTTCTGACATCGAAGCTAACGCCCTCTTagagag                                                                                                                                                                                                                                                       <  1:398123‑M2/97‑1 (MQ=255)`  
                                                     `agtaatcagcaaggcgatatgcctaaccttgtcctcgttcgtattctcaagtgccgctttactggtgatactggtatcgctggctacatggaatacaacaaggaaaccggatggcttgaaccatcaagttactcaggggaagaagagtcacactcagagtcaacagactggtccaacgacactgacttctgacaggattcttgaCAGTTGTTTCATATGAAGAGATTGTTAAGTCACGATAATC‑AATAGGAGAAATCAATATGATCGTTTCTGACATCGAAGCTAACGCCCTCTTagagag                                                                                                                                                                                                                                                       <  1:269018‑M2/97‑1 (MQ=255)`  
                                                      `gtaatcagcaaggcgatatgcctaaccttgtcctcgttcgtattctcaagtgccgctttactggtgatactggtatcgctggctacatggaatacaacaaggaaaccggatggcttgaaccatcaagttactcagggaaagaagagtcacactcagagtcaacagactggtccaacgacactgacttctgacaggattcttgaCAGTTGTTTCATATGAAGAGATTGTTAAGTCACGATAATC‑AATAGGAGAAATCAATATGATCGTTTCTGACATCGAAGCTAACGCCCTcttagagctg                                                                                                                                                                                                                                                      >  1:231204‑M2/204‑298 (MQ=255)`  
                                                        `gTCATAAGGTGTTCGATAAGGACACTATGACCCACATCAAGGCTGGTGATTGGGTTAAGGTTGACATGGGAGTTGTTGGTGGATACTGCTACGTCCGCTCAGTTAGTGGCAAATATGCACAAGTGTCATACATCACAGGTGTTACTCCACGCGGTGCAATCGTTGCCTATAAGACCAACATGATTCACACAGGTTTCTTGACAGTTGTTTCATATGAAGAGATTGTTAAGTCACGATAATC‑AATAGGAGAAATCAATATGATCGTTTCTGACATCGAAGCTAACGCCCTCTTAGAGAGCGt                                                                                                                                                                                                                                                    >  1:475434/1‑301 (MQ=255)`  
                                                           `gtataagagacagtgcctaatcttgtccacgtgcgtattctcaagtgccgctttactggtgatactgttatcgctagctacatggaatacaacaaggaaaccggatggcttgaaccatcaagttactcaggggaagaagagtcacactcatagtcaacagactggtccaacgacactgacttctgacaggattcttgaCAGTTGTTTCATATGAAGAGATTGTTAAGTCACGATAATC‑AATAGGAGAAATCAATATGATCGTTTCTGACATCGAAGCTAACGCCCTCTTAGAGAGCGTCAc                                                                                                                                                                                                                                                 <  1:108723‑M2/103‑1 (MQ=255)`  
                                                           `cagcaagtcgatatgcctaaccttgtcctcgttcgtagtctcaagtgccgctttactggtgatactggtatctctggctacatggaatacaacaaggaaaccggatggcttgaaccatcaagttactcaggggaagaagagtcacactcagagtcaacagactggtccaacgacactgacttctgacaggattcttgaCAGTTGTTTCATATGAAGAGATTGTTAAGTCACGATAATC‑AATAGGAGAAATCAATATGATCGTTTCTGACATCGAAGCTAACGCCCTCTTAGAGAGCGTCAc                                                                                                                                                                                                                                                 <  1:213825‑M2/103‑1 (MQ=255)`  
                                                               `ggTGTTCGATAAGGACACTATGACCCACATCAAGGCTGGTGATTGGGTTAAGGTTGACATGGGAGTTGTTGGTGGATACGGCTACGTCCGCTAAGTTAGTGGCAAATATGCACAAGTGTCATACATCACAGGTGTTACTCCACGCGGTGCAATCGTTGCCGATAAGACCAACATGATTCACACAGGTTTCTTGACAGTTGTTTCATATGAAGAGATTGTTAAGTCACGATAATC‑AATAGGAGAAATCAATATGATAGTTTCTGACATCGAAGCTAACGCCCTCTTAGAGAGCGTAACTact                                                                                                                                                                                                                                             >  1:36146/1‑299 (MQ=255)`  
                                                                  `gcgatatgcctaaccttgtcctcgttcgtattctcaagtgccgctttactggtgatactggtatcgctggctacatggaatacaacaaggaaaccggatggcttgaaccatcaagttactcaggggaagaagagtcacactcagagtcaacagactggtccaacgacactgacttctgacaggattcttgaCAGTTGTTTCATATGAAGAGATTGTTAAGTCACGATAATC‑AATAGGAGAAATCAATATGATCGTTTCTGACATCGAagctaacgccctcttagagctgtctcttatacac                                                                                                                                                                                                                                          >  1:235062‑M2/192‑286 (MQ=255)`  
                                                                   `ggatatgcctaaccttgtcctcgttcgtattctcaagtgccgctttactggttatactggtatcgctggctacatggaatacaacaaggaaaccggatggcttgaaccatcaagttactcagggtaagaagagtcacactcagagtcaacagactggtccaacgacactgacttctgacaggattcttgaCAGTTGTTTCATATGAATAGATTGTTAAGTCACGATAATC‑AATAGGAGAAATCAATATGATCGTTTCTGACATCGAAGCTAACGCCCTCTTAGAGAGCGTCACTAAGTTcc                                                                                                                                                                                                                                         <  1:429659‑M2/111‑1 (MQ=255)`  
                                                                       `atgcctaaccttgtcctcgttcgtattctcaattgccgctttactggtgatactggtatcgctggctacatggaatacaacaaggaaaccggatggcttgaaccatcaagttactcaggggaagaagagtcacactcagagtcaacagactggtccaacgacactgacttctgacaggattcttgaCAGTTGTTTCATATGAAGAGATTGTTAAGTCACGATAATC‑AATAGGAGAAATCAATATGATCGTTTCTGACATCGAAGCTAACGCCCTCTTAGATAGCGTCACTAAGTTCCACTg                                                                                                                                                                                                                                     <  1:59027‑M2/115‑1 (MQ=255)`  
                                                                         `gcctaaccttgtcctcgttcgtattctcaagtgccgctttactggtgatactggtatcgctggctacatggaatacaacaaggaaaccggatggcttgaaccatcaagttactcaggggaagaagagtcacactcagagtcaacagactggtccaacgacactgacttctgacaggattcttgaCAGTTGTTTCATATGAAGAGATTGTTAAGTCACGATAATC‑AATAGGAGAAATCAATATGATCGTTTCTGACATCGAAGCTAACGCCCTCTTAGAGAGCGTCACTAAGTTCCACTGCg                                                                                                                                                                                                                                   >  1:374589‑M2/185‑301 (MQ=255)`  
                                                                         `gcctaaccttgtcctcgttcgtattctcaagtgccgctttactggtgatactggtatcgctggctacatggaatacaacaaggaaaccggatggcttgaaccatcaagttactcaggggaagaagagtcacactcagagtcaacagactggtccaacgacactgacttctgacaggattcttgaCAGTTGTTTCATATGAAGAGATTGTTAAGTCACGATAATC‑AATAGGAGAAATCAATATGATCGTTTCTGACATCGAAGCTAACGACCTCTTAGAGAGCGTCACTAAGTTCCACTGCg                                                                                                                                                                                                                                   >  1:366871‑M2/185‑301 (MQ=255)`  
                                                                                       `gcgtatgtattctcaagtgccgctttactggtgatactggtatcgctggctacatggaatacaacaaggaaaccggatggcttgaaccatcaagttactcaggggaagaagagtcacactcagagtcaacagactggtccaacgatactgactactgacaggattcttgaCAGTTGTTTCATATGAAGAGATTGTTAAGTCACGATAATC‑AATAGGAGAAATCAATATGATCGTTTCTGACATCGAAGCTAACGCCCTCTTAGAGAGCGTCACTAAGTTCCACTGCGGGGTTATCTACGac                                                                                                                                                                                                                     <  1:386328‑M2/131‑1 (MQ=255)`  
                                                                                        `cgttcgtattctcaagtgccgctttactggtgatactggtatcgctggctacatggaatacaacaaggaaaccggatggcttgaaccatcaagttactcaggggaagaagagtcacactcagagtcaacagactggtccaacgacactgacttctgacaggattcttgaCAGTTGTTTCATATGAAGAGATTGTTAAGTCACGATAATC‑AATAGGAGAAATCAATATGATCGTTTCTGACATCGAAGCTAACGCACTCTTAGAGAGCGTAACTAAGTTCCACTGCGGGGTTATCTACGact                                                                                                                                                                                                                    >  1:190811‑M2/170‑301 (MQ=255)`  
                                                                                          `caTCAAGGCTGGTTATTGGGTTAAGGTTGACATGGGAGTTGTTGGTGGATACGGCTACGTCCGCTCAGTTAGTGGCAAATATGCACAAGTGTCATACATCACAGGTGTTACTCCACGCGGTGCAATCGTTGCCGATAAGACCAACATGATTCACACAGGTTTCTTGACAGTTGTTTCATATGAAGAGATTGTTAAGTCACGATAATC‑AATAGTAGAAATCAATATGATCGTTTCTGACATCGAAGCTAACGCCCTCTTAGAGAGCGTCACTAAGTTCCACTGCGGGGTTATCTACGactaa                                                                                                                                                                                                                  >  1:206286/1‑300 (MQ=255)`  
                                                                                               `attctcaagtgccgctttacgggtgatactggtatcgctggctacatggaatacaacaaggaaaccggatggcttgaaccatcaagttactcaggggaagaagagtcacactcagagtcaacagactggtccaacgacactgacttctgacaggattcttgaCAGTTGTTTCATATGAAGAGATTGTTAAGTCACGATAATC‑AATAGGAGAAATCAATATGATCGTTTCTGACATCGAAGCTAACGCCCTCTTAGAGAGCGTCACTAAGTTCCACTGCGGGGTTATCTACGACTACTacac                                                                                                                                                                                                             <  1:138389‑M2/139‑1 (MQ=255)`  
                                                                                                  `gagacaggccgctttactggtgatactggtatcgctggctacatggaatacaacaaggaaaccggatggcttgaaccatcaagttactcaggggaagaagagtcacactcagagtcaacagactggtccaacgacactgacttctgacaggattcttgaCAGTTGTTTCATATGAAGAGATTGTTAAGTCACGATAATC‑AATAGGAGAAATCAATATGATCGTTTCTGACATCGAAGCTAACGCCCTCTTAGAGAGCGTCACTAAGTTCCACTGCGGGGTTATCTACGACTACTCCACCGc                                                                                                                                                                                                          <  1:378196‑M2/142‑1 (MQ=255)`  
                                                                                                  `ctcaagtgccgctttactggtgatactggtatcgctggctacatggaatacaacaaggaaaccggatggcttgaaccatcaagttactaaggggaagaagagtcacactcagagtcaacagactggtccaacgacactgacatctgacaggattcttgaCAGTTGTTTCATATGAAGAGATTGTTAAGTCACGATAATC‑AATAGGAGAAATCAATATGATCGTTTCTGACATCGAAGCTAACGCCCTCTTAGAGAGCGTCACTCAGTTCCACTGCGGGGTTATCTACGACTACTACACcga                                                                                                                                                                                                          >  1:53843‑M2/160‑300 (MQ=255)`  
                                                                                                            `gctttactggtgatactggtatcgctggctacatggaatacaacaaggaaaccggatggcttgaaccatcaagttactcaggggaagaagagtcacactcagagtcaacagactggtccaacgacactgacgtctgacaggtttcttgaCAGTTGTTTCATATGAAGAGATTGTTAAGTCACGATAATC‑AATAGGAGAAATCAATATGATCGTTTCTGACATCGAAGCTAACGACCTCTTAGAGAGCGTCACTAAGTTCCACTGCGGGGTTATCTACGACTACTCCACCGCTGAGTACGTa                                                                                                                                                                                                >  1:442982‑M2/150‑301 (MQ=255)`  
                                                                                                              `gagacaggtgatactggtatcgctggctacatggaatacaacaaggaaaccggatggcttgaaccatcaagttactcaggggtagaagagtcacactcagagtcaacagactggtccaacgacactgacttctgacaggattcttgaCAGTTGTTTCATATGAAGAGATTGTTAAGTCACGATAATC‑AATAGGAGAAATCAATATGATCGTTTCTGACATCGAAGCTAACGCCCTCTTAGAGAGCGTCACTAAGTTCCACTGCGGGGTTATCTACGACTACTCCACCGCTGAGTACGTAAg                                                                                                                                                                                              <  1:197355‑M2/154‑1 (MQ=255)`  
                                                                                                                         `tactggtatcgctggctacatggaatacaacaaggaaaccggatggcttgaaccatcaagttactaaggggaagaagagtcacactcagagtcaacagactggtccaacgacactgacttctgacaggattcttgaCAGTTGTTTCATATGAAGAGATTGTTACGTCCCGATAATC‑AATAGGAGAAATCAATATGATCGTTTCTGAACTCGAAGCTAACGCCCTCTTAGAGAGCTTCACTAAGTTCCACTGCGGGGTTATCTACGACTACTCCCCGGCTGAGTACGTAAGCTACCGTCcgg                                                                                                                                                                                   >  1:183473‑M2/137‑300 (MQ=255)`  
                                                                                                                                `ttgttgGTGGATACGGCTACGTCCGCTAAGTTAGTTGCAAATATGCACAAGTGTCATACATCACAGGTGTTACTCCACGCGGTGCAATCGTTGCCGATAAGACCAACATGATTCACACAGGTTTCTTGACAGTTGTTTCATATGAAGAGATTGTTAAGTCACGATAATC‑AATAGGAGAAATCAATATGATCGTTTCTGACATCGAAGCTAACGACCTCTTAGAGAGCGTCACTAAGTTCCACTGCGGGGTTATCTACGACTACTCCACCGCTGAGTACGTAAGCTACCGTCCGAGTGACtt                                                                                                                                                                            <  1:479338/301‑1 (MQ=255)`  
                                                                                                                                        `ctacatggaatacaacaaggaaaccggatggcttgaaccatcaagttactcaggggaagaagagtcacactcagagtcaacagactggtccaacgacactgacttctgacaggattcttgaCAGTTGTTTCATATGAAGAGATTGTTAAGTCACGATAATC‑AATAGGAGAAATCAATATGATCGTTTCTGACATCGAAGCTAACGCCCTCTTAGAGAGCGTCACTAAGTTCCACTGCGGGGTTATCTACGACTACTCCACCGCTGAGTACGTAAGCTACCGTCCGAGTGACTTCGGTGCGt                                                                                                                                                                    >  1:115019‑M2/122‑301 (MQ=255)`  
                                                                                                                                           `aatggaatacaacaaggaaaacggatgggttgaaccatcaagtaactcagtagaagaagagtcacactcagagaaaacagacagatccaaagaaactgacttctgacaggattcttgaCAGTTGTTGCATATGAAGAGATGGTGAAGTAACGATAATC‑AATAGTAGAAATCAATATAATCGTTTCTGAAATCGAAGATAACGCCATCTTAGAGAGCGTCACTAAGTTCCACTGCGGGGTTATCTACGACTACTCCACCGCTGAGTATGTAAGCTACCGTCCGAGTGACTTCGGTGCGTATc                                                                                                                                                                 <  1:88311‑M2/183‑1 (MQ=255)`  
                                                                                                                                             `tggaatacaacaaggaaaccggatggcttgaaccatcaagttagtcaggggaagaagagtcacactcagagtcaacagactggtccaacgacactgacttctgacaggattcttgaCAGTTGTTTCATATGAAGAGATTGTTAAGTCACGATAATC‑AATAGGAGAAATCAATATGATCGTTTCTGACATCGAAGCTAACGCCCTCTTAGAGAGCGTCACTAAGTTCCACTGCGGGGTTATCTACGACTACTCCACCGCTGAGTACGTAAGCTACCGTCCGAGTGACTTCGGTGCGTATCTg                                                                                                                                                               <  1:246298‑M2/185‑1 (MQ=255)`  
                                                                                                                                               `gaatacaacaaggaaaccggatggcttgaaccatcaagttactcaggggaagaagagtcacactcagagtcaacagactggtccaacgacactgacttctgacaggattcttgaCAGTTGTTTCATATGAAGAGATTGTTAAGTCACTATAATC‑AATAGGAGAAATCAATATGATCGTTTCTGACATCGAAGCTAACGCCCTCTTAGAGAGCGTCACTAAGTTCCACTGCGGGGTTATCTACGACTACTCCACCGCTGAGTACGTAAGCTACCGTCCGAGTGACTTCGGTGCGTTTCTGGa                                                                                                                                                             >  1:504671‑M2/115‑301 (MQ=255)`  
                                                                                                                                               `gaatacaacaaggaaaccggatggcttgaaccatcaagttactcaggggaagaagagtcacactcagagtcaacagactggtccaacgacactgacttctgacaggattcttgaCAGTTGTTTCATATGAAGAGATTGTTAAGTCACGATAATC‑AATAGGAGAAATCAATATGATCGTTTCTGACATCGAAGCTAACGCCCTCTTAGAGAGCGTCACTAAGTTCCACTGCGGGGTTATCTACTACTACTCCACCGCTGAGTACGTAAGCTACCGTCCGAGTGACTTCGGTGCGTATCTGGa                                                                                                                                                             >  1:393386‑M2/115‑301 (MQ=255)`  
                                                                                                                                               `gaatacaacaaggaaaccggatggcttgaaccatcaagttactcaggggaagaagagtcacactcagagtcaacagactggtccaacgacactgacttctgacaggattcttgaCAGTTGTTTCATATGAAGAGATTGTTAAGTCACGATAATC‑AAAAGGAGAAATCAATATGATCGTTTCTGACATCGAAGCTAACGCCCTCTTAGAGAGCGTCACTAAGTTCCACTGCGGGGTTATCTACGACTACTCCACCGCTGAGTACGTAAGCTACCGTCCGAGTGACTTCGGTGCGTATCtggg                                                                                                                                                             >  1:370704‑M2/115‑300 (MQ=255)`  
                                                                                                                                               `gaataaaaaaaggaaaccggatggcgtgaaccatcaaggtacacaggggaagaagagtcaaactcagatgcaacagactggtccaacgacactgactgctgacaggattcttgaCAGTTGTTTCATATGAAGAGATTGTTAAGTCACGATAATC‑AATAGGAGAAATCAATATGATCGTTTCTGACATAGAAGCTAACGCCCTCTTAGAGAGCGTCACTAAGTTCCACTGCGGGGTTATTTACGACTACTCCACCGCTGAGTACGTAAGCTACCGTCCGAGTGACTTCGGTGCGTATCTGGa                                                                                                                                                             <  1:84260‑M2/187‑1 (MQ=255)`  
                                                                                                                                                   `acaacaaggaaaccggatggcttgaaccatcaagttactcaggggaagaagagtcacactcagagtcaacagactggtccaacgacactgacttctgacaggattcttgaCAGTTGTTTCATATGAAGAGATTGTTAAGTCACGATAATC‑AATAGGAGAAATCAATATGATCTTTTCGGACATCGAAGCTAACGCACTCTTCGAGAGCGTCACTAAGTTCCACTGCGGGGTTATCTACGACTACTCCACCGCAGAGTACGTAAGCTACCGTCCGAGTAACTTCGTTGCGTCTATGGATgcg                                                                                                                                                         >  1:65170‑M2/111‑301 (MQ=255)`  
                                                                                                                                                   `acaacaaggaaaccggatggcttgaaccatcaagttactcaggggaagaagagtcacactcagagtcaacagactggtacaacgacactgacttctgagaggattcttgaCAGTTGTTTCATATGAAGAGATTGTTAAGTCACGATAATC‑AATAGGAGAAATCAATATGATCGTTTCTGATATCGAAGCTAACGCCCTCTTAGAGAGCGTCACTAAGTGCCACTGCTGGGGTATCTACGATTACTCCAACGCTGAGTGCGTAAGCTAGCGTCCGAGCGCCTTCGGTGCGTATCTGTATgcg                                                                                                                                                         >  1:410942‑M2/111‑301 (MQ=255)`  
                                                                                                                                                        `aaggaaaccggatggcttgaaccatcaagttactcaggggaagaagagtcacactcagagtcaacagactggtccaacgacactgacttctgacaggattcttgaCAGTTGTTTCATATGAAGAGATTGTTAAGTCACGATAATC‑AATAGGAGAAATCAATATGATCGTTTCTGACATCGAAGCTAACGCCCTCTTAGAGAGCGTCACTAAGTTCCACTGCGGGGTTATCTACGACTACTCCACCGCTGAGTACGTAAGCTACCGTCCGAGTGACTTCGGTGCGTTACTGGATGCGCtggg                                                                                                                                                    >  1:271726‑M2/106‑300 (MQ=255)`  
                                                                                                                                                                     `ggcttgaaccatcaagttactcaggggaagaagagtcacactcagagtcaacagactggtccaacgacactgacttctgacaggattcttgaCAGTTGTTTCATATGAAGAGATTGTTAAGTCACGATAATC‑AATAGGAGAAATCAATATGATCGTTTCTGACATCGAAGCTAACGCCCTCTTAGAGAGCGTCACTAAGTTCCACTGCGGGGTTATCTACGACTACTCCACCGCTGAGTACGTAAGCTACCGTCCGAGTGACTTCGGTGCGTATCTGGATGCGCTTGAAGCCGAGGTTgcc                                                                                                                                       >  1:172881‑M2/93‑300 (MQ=255)`  
                                                                                                                                                                      `gcttgaaccatcaagttactcaggggaagaagagtcacactcagagtcaacagactggtccaacgacactgacttctgacaggattcttgaCAGTTGTTTCATATGAAGAGATTGTTAAGTCACGATAATC‑AATAGGAGAAATCAATATGATCGTTTCTGACATCGAAGCTAACGCCCTCTTAGAGAGCGTCACTAAGTTCCACTGCGGGGTTATCTACGACTACTCCACCGCTGAGTACGTAAGCTACCGTCCGAGTGACTTCGGTGCGTATCTGGATGCGCTGGAAGCCGAGGTTGCAc                                                                                                                                      <  1:445949‑M2/210‑1 (MQ=255)`  
                                                                                                                                                                                     `ttactcaggggaagaagagtcacactcagagtcaacagactggtccaacgacactgacttctgacaggattcttgaCAGTTGTTTCATATGAAGAGATTGTTAAGTCACGATAATC‑AATAGGAGAAATCAATATGATCGTTTCTGACATCGAAGCTAACGCCCTCTTAGAGAGCGTCACTAAGTTCCACTGCGGGGTTATCTACGACTACTCCACCGCTGAGTACGTAAGCTACCGTCCGAGTGACTTCGGTGCGTATCTGGATGCGCTGGAAGCCGAGGTTGCACGAGGCGGTCTTATtg                                                                                                                       <  1:222330‑M2/225‑1 (MQ=255)`  
                                                                                                                                                                                     `cagcacaggggaagaagagtcacactcagagtcaacagactggtccaacgacactgacttctgacaggattcttgaCAGTTGTTTCATATGAAGAGATTGTTAAGTCACGATAATC‑AATAGGAGAAATCAATATGATCGTTTCTGACATCGAAGCTAACGCCCTCTTAGAGAGCGTCACTAAGTTCCACTGCGGGTTTATCTACGACTACTCCACCGCTGAGTACGTAAGCTACCGTCCGAGTGACTTCGGTGCGTATCTGGATGCGCTGGAAGCCGAGGTTGCACGAGGCGGTCTTATtg                                                                                                                       <  1:112590‑M2/225‑1 (MQ=255)`  
                                                                                                                                                                                          `acaggtaagaagattcacactgatagtcagcagactggtcccacgacattgatttctgtcaggattattgaCATTTGTTTAATATGAAGAGATTGTTAAGTCACGTTAATC‑AATAGGAGAAATCAATATGATCGTTTCTGACATCGAAGCTAACGCCCTCTTAGATAGCGTCACTAAGTTCCACTGCGGGGTTATCTACGACTACTCCACCGCTGAGTACGTAAGCTACCGTCCGAGTGACTTCGGTGCGTATCTGGATGCGCTGGAAGCCGAGGTTGCACGAGGCGGTCTTATTGTGTTc                                                                                                                  <  1:67999‑M2/230‑1 (MQ=255)`  
                                                                                                                                                                                            `ggggaagaagagtcacactcagagtcaacagactggtccaacgacactgacttctgacaggattcttgaCAGTTGTTTCATATGAAGAGATTGTTAAGTCACGATAATC‑AATAGGAGAAATCAATATGATCGTTTCTGACATCGAAGCTAACGCCCTCTTAGAGAGCGTCACTAAGTTCCACTGCGGGGTTATCTACGACTACTCCACCGCTGAGTACGTAAGCTACCGTCCGAGTGACTTCGTTGCGTATCCGGATGCGCTGGAAGCCGAGGTTGCACGAGGCGGTCTTATTGTGTtccc                                                                                                                >  1:318614‑M2/70‑300 (MQ=255)`  
                                                                                                                                                                                                  `gtgtTACTCCACGCGGTGCAATCGTTGCCGATAAGACCAACATGATTCACACAGGTTTCTTGACAGTTGTTTCATATGAAGAGATTGTTAAGTCACGATAATC‑AATAGGAGAAACCATTATGATCGTTTCTGACATCGAAGCTAACGCCCTCTTAGAGAGCGTCACTAAGTTCCACTGCGGGGTTATCTACGACTACTCCACCGCTGAGTACGTAAGCTACCGTCCGAGTGACTTCGGTGCGTATCTGGATGCGCTGGAAGCCGAGGTTGCACGAGGCGGTCTTCTTGTGTTCCACAACgg                                                                                                          >  1:203284/1‑301 (MQ=255)`  
                                                                                                                                                                                                       `aCTCCACGCGGTGCAATCGTTGCCGATAAGACCAACATGATTCACACAGGTTTCTTGACAGTTGTTTCATATGAAGAGATTGTTAAGTCACGATAATC‑AATAGGAGAAATCAATATGATCGTTTCTGACATCGAAGCTAACGCCCTCTTAGAGAGCGTCACTAAGTTCCACTGCGGGGTTATCTACGACTACTCCACCGCTGAGTACGTAAGCTACCGTCCGAGTGACTTCGGTGCGTATCTGGATGCGCTGGAAGCCGAGGTTGCACGAGGCGGTCTTATTGTGTTCCACAACGGTcaca                                                                                                     >  1:100278/1‑301 (MQ=255)`  
                                                                                                                                                                                                         `agcactcagagtcaacagactggtccaacgacactgacttctgacaggattcttgaCAGTTGTTTCATATGAAGAGATTGTTAAGTCACGATAATC‑AATAGTAGAAATCAATATGATCGTTTCTGACATCGAAGCTAACGCCCTCTTAGAGAGCGTCACTAAGTTCCACTGCGGGGTTATCTACGACTACTCCACCGCTGAGTACGTAAGCTACCGTCCGAGTGACTTCGGTGCGTATCTGGATGCGCTGGAAGCCGAGGTTGCACGAGGCGGTCTTATTGTGTTCCACAACGGTCACAAg                                                                                                   <  1:406992‑M2/245‑1 (MQ=255)`  
                                                                                                                                                                                                             `ctcagcgtcaacagactggtccaacgacactgacttctgacaggattcttgaCAGTTGTTTCATATGAAGAGATTGTTAAGTCACGATAATC‑ACTAGGAGAAATCAATATGATCGTTTCTGACATCGAAGCTAACGCCCTCTTAGAGAGCGTCACTAATTTCCACTGCCGGGTTATCTACGACTCCTCTACCGCTGACTACGTAAGCTACCGTCCGACTGACTTCGGTGCGTATCAGGATGCGCTGGAAGCCGAGGTTGCACGCGGCGGCCTTATTGTGTTCAACAACGGCACCAAGTATg                                                                                               >  1:162659‑M2/53‑301 (MQ=255)`  
                                                                                                                                                                                                                `tgggctcggagatgtgtataagagacagtacttctgacaggattctttaCAGTTGTTTCATATGAAGAGATTGTTAAGTCACGATAATC‑AATAGGAGAAATCAATATGATCGTTTCTGACATCGAAGCTAACGCCCTCTTAGAGAGCGTCACTAAGTTCCACTGCGGGGTTATCTACGACTACTCCACCGCTGAGTACGTAAGCTACCGTCCGAGTGACTTCGGTGCGTATCTGGATGCGCTGGAAGCCGAGGTTGCACGAGGCGGTCTTATTGTGTTCCACAACGGTAACAAGTATGACg                                                                                            <  1:16617‑M2/252‑1 (MQ=255)`  
                                                                                                                                                                                                                `agagtcaacagactggtccaacgacactgacttctgacaggattcttgaAAGTTGTTTCATATGAATAGATTGTTAAGTCACGATAATC‑AATAGGAGAAATCAATATGATCGTTTCTGACATCGAAGCTAACGCCCTCTTAGAGAGCGTCACTAAGTTCCACTGCGGGGTT‑TCTACGACTACTCCACCGCTGAGTACGTAAGCTACCGTCCGAGTGACTTCGGTGCGTATCTGGATGCGCTGGAAGCCGAGGTTGCACGAGGCGGTCTTATTGTGTTCCACAACGGTCACAAGTATGACGt                                                                                           <  1:463242‑M2/252‑1 (MQ=255)`  
                                                                                                                                                                                                                 `gtgtataagagacagtccaacgacactgacttctgacaggattcttgaCAGTTGTTTCATATGAAGAGATTGTTAAGTCACGATAATC‑AATAGGAGAAATCAATATGATCGTTTCTGACATCGAAGCTAACGCCCTCTTAGAGAGCGTCACTAAGTTCCACTGTGGGGTTATCTACGACTACTCCACCGCTGAGTACGTAAGCTACCGTCCGAGTGACTTCGGTGCGTATCTGGATGCGCTGGAAGCCGAGGTTGCACGAGGCGGTCTTATTGTGTTCCACAACGGTCACAAGTATGACGt                                                                                           <  1:240838‑M2/253‑1 (MQ=255)`  
                                                                                                                                                                                                                 `gagtcaacagactggtccaacgacactgacttctgacaggattcttgaCAGTTGTTTCATATGAAGAGATTGTTAAGTCACGATAATC‑AATAGGAGAAATCAATATGATCGTTTCTGACATCGAAGCTAACGCCCTCTTAGAGAGCGTCACTAAGTTCCACTGCGGGGTTATCTACGACTACTCCACCGCTGAGTATGTAAGCTACCGTCCGAGTGACTTCGGTGCGTATCTGGATGCGCTGGAAGCCGAGGTTGCACGAGGCGGTCTTATTGTGTTCCACAACGGTCACAAGTATGACGt                                                                                           <  1:29525‑M2/253‑1 (MQ=255)`  
                                                                                                                                                                                                                 `gTGCAATCGTTGCCGTTAAGACCAACATGATTCAGTCAGGTTTCTAGACAGTTGTTTCATATGAAGAGATGATTAAGTCACGATAATC‑AATAGGAGAAATCAATATGATCGTTTCTGACATCGAAGCTAACGCCCTCTTAGAGAGCGTCACTAAGTTCCACTGCGGGGTTATCTACGACTACTCCACCGCTGAGTACGTAAGCTACCGTCCGAGTGACTTCGGTGCGTATCTGGATGCGCTGGAAGCCGAGGTTGCACGAGGCGGTCTTATTGTGTTCCACAACGGTCACAAGTATGACGt                                                                                           <  1:449264/301‑1 (MQ=255)`  
                                                                                                                                                                                                                      `agatacagggtccaacgacacggacttctgacaggtttctggaCAGTTGTTTCATATGAATAGATTGTCAAGTCACGATAATC‑AATAGGAGAAATCAATATGATCGTTTCTGACAGCGAAGCTAACGCCCTCTTAGAGAGCGTCACTAAGTTCCACTGCGGGGTTATCTACGACTACTCCACCGCTGAGTACGTAAGCTACCGTCCGAGTGACTTCGGTGCGTATCTGGATGCGCTGGAAGCCGAGGTTGCACGAGGCGGTCTTATTGTGTTCCACAACGGTCACAAGTATGACGTTCCTg                                                                                      <  1:62053‑M2/258‑1 (MQ=255)`  
                                                                                                                                                                                                                      `aTCGTTGCCGATAAGACCAACATTATTCACACAGGTTTCTTGACAGTTGTTTCATATGAAGAGATTGTTAAGTCACGATAATC‑AATAGGAGAAATCAATATGATCGTTTCTGACATCGAAGCTAACGCCCTCTTAGAGAGCGTCACTAAGTTCCACTGCGGGGTTATCTACGACTACTCCACCGCTGAGTACGTAAGCTACCGTCCGAGTGACTTCGGTGCGTATCTGGATGCGCTGGAAGCCGAGGTTGCACGAGGCGGTCTTATTGTGTTCCACAACGGTCACAAGTATGActgtctct                                                                                      >  1:14746/1‑294 (MQ=255)`  
                                                                                                                                                                                                                         `agactggtccaacgacactgacttctgacaggattcttgaCAGTTGTTTCATATGAAGAGATTGTTAAGTCACGATAATC‑AATAGGAGAAATCAATATGATCGTTTCTGACATCGAAGCTAACGCCCTCTTAGAGAGCGTCACTAAGTTCCACTGCGGGGTTATCTACGACTACTCCACCGCTGAGTACGTAAGCTACCGTCCGAGTGACTTCGGTGCGTATCTGGATGCGCTGGAAGCCGAGGTTGCACGAGGCGGTCTTATTGTGTTCCACAACGGTCACAAGTATGACGTTCCTGCAt                                                                                   <  1:287854‑M2/261‑1 (MQ=255)`  
                                                                                                                                                                                                                         `aaacaggtccaacgacactgacttctgacaggattcttgaCAGTTGTTTCATATGAAGAGATTGTTAAGTCACGATAATC‑AATAGGAGAAATCAATATGATCGTTTCTGACATCGAAGCTAACGCCCTCTTAGAGAGCGTCACTACGTTCCACTGCGGGGTTATCTACGACTACTCCACCGCTGAGTACGTAAGCTACCGTCAGAGTGACTTCGGTGCGTATCTGGCTGCGCTGGAAGCCGAGGTTGCACGAGGCGGTCTTATTGTGTTCCACAACGGTCACAAGTATGACGTTCCTGCAt                                                                                   <  1:77448‑M2/261‑1 (MQ=255)`  
                                                                                                                                                                                                                                        `aaCATGATGCACACAGGTTTCTTGACAGTTGTTTCATAAGAAGAGATTGTTAAGTAACGATAATC‑AATAGGAGAAATCAATAGGATCGTTTCTGACATCGAAGCTAACGCCCGCTTAGAGAGCGTCACTAAGTTCCACTGCGGGGTTATCTACGACTACTCCACCGCTGAGTACGTAAGCTACCGTCCGAGTGACTTCGGTGCGTATCTGGATGCGCTGGAAGCCGAGGTTGCACGAGGCGGTCTTATTGTGTTCCACAACGGTCACAAGTATGACGTTCCTGCATTGACCAAACTGGCaa                                                                    <  1:351010/301‑1 (MQ=255)`  
                                                                                                                                                                                                                                           `gtccaacgacactgacttctgaCAGTTGTTTCATATGAAGAGATTGTTAAGTCACGATAATC‑AATAGGAGAAATCAATATTATCGTTTCTGACATCGAAGCTAACGCCCTCTTAGAGAGCGTCACTAAGTTCCACTGCGGGGTTATCTACGACTACTCCACCGCTGAGTACGTAAGCTACCGTCCGAGTGACTTCGGTGCGTATCTGGATGCGCTGGAAGCCGAGGTTGCACGAGGCGGTCTTATTGTGTTCCACAACGGTCACAAGTATGACGTTCCTGCATTGACCAAACTGGCAAAGt                                                                 >  1:91382‑M2/23‑301 (MQ=255)`  
                                                                                                                                                                                                                                            `gacttctgacaggattcttgaCAGTTGTTTCATATGAAGAGATTGTTAAGTCACGATAATC‑AATAGGAGAAATCAATATGATCGTTTCTGACATCGAAGCTAACGCCCTCTTAGAGAGCGTCACTAAGTTCCACTGCGGGGTTATCTACGACTACTCCACCGCTGAGTACGTAAGCTACCGTCCGAGTGACTTCGGTGCGTATCTGGATGCGCTGGAATCCGAGGTTGCACGAGGCGGTCTTATtgtgttccacaacggtcacaagtatgacgctgtctcttatacacatctccgagcccc                                                                >  1:215879‑M2/22‑273 (MQ=255)`  
                                                                                                                                                                                                                                            `gacttctgacaggattcttgaCAGTTGTTTCATATGAAGAGATTGTTAAGTCACGATAATC‑AATAGGAGAAAGCAATATGATCGTTTCTGACATCGAAGCTAACTCCCTCTTAGAGAGCGTCACTAAGTTCCACTGCGGGGTTATCTACGACTACTCCACCGCTGAGTACGTAAGCTACCGTCCGAGTGACTTCGGTGCGTATCTGGATGCGCTGTAAGCCGAGGTTGCACGAGGCGGTCTTATTGTGTTCCACAACGgtcacaagtatgacgtttctgcctgtctcttatacacatctcc                                                                >  1:455415‑M2/22‑280 (MQ=255)`  
                                                                                                                                                                                                                                                   `gacaggattcttgaCAGTTGTTTCATATGAAGAGATTGTTAAGTCACGATAATC‑AATAGGAGAAATCAATATGATCGTTTCTGACATCGAAGCTAACGCCCTCTTAGAGAGCGTCACTAAGTTCCACTGCGGGGTTATCTACGACTACTCTACCGCTGAGTACGTAAGCTACAGTCCGAGTGACTTCGGTGCGTATCTGGATGCGCTGGAAGCCGAGGTTGCACGCGGCGGTCTTATTGTGTTCCAAAACGGTCACAAGTATGACGTTCCTGCATTTACCAAACTGGCAAAGTTGCAATTg                                                         >  1:467139‑M2/15‑301 (MQ=255)`  
                                                                                                                                                                                                                                                       `ggattcttgaCAGTTGTTTCATATGAAGAGATTGTTAAGTCACGATAATC‑AATAGGAGAAATCAATATGATCGTTTCTGACATCGAAGCTAACGCCCTCTTAGAGAGCGTCACTAAGTTCCACTGCGGGGTTATCTACGACTACTCCACCGCTGAGTACGTAAGCTACCGTCCGAGTGACTTCGGTGCGTATCTGGATGCGCTGGAAGCCGAGGTTGCACGAGGCGGTCTTATTGTGTTCCACAACGGTCACAAGTATGACGttcctgcattgaccaaactgctgtctcttatacacatct                                                     >  1:121696‑M2/11‑282 (MQ=255)`  
                                                                                                                                                                                                                                                       `ggattcttgaCAGTTGTTTCATATGAAGAGATTGTTAAGTCACGATAATC‑AATAGGAGAAATCAATATGATCGTTTCTGACATCGAAGCTAACGCCCTCTTAGAGAGCGTCACTAAGTTCCACTGCGGGGTTATCTACGACTACTCCACCGCTGAGTACGTAAGCTACCGTCCGAGTGACTTCGGTGCGTATCTGGATGCGCTGGAAGCCGAGGTTGCACGAGGCGGCCTTATTGTGTTCCACAACGGTCACAAGTATAACGTTCCTGCATTGACCAAACTGGCAAAGTTGCAATTGAAcc                                                     >  1:266422‑M2/11‑301 (MQ=255)`  
                                                                                                                                                                                                                                                        `gattcttgaCAGTTGTTTCATATGAAGAGATTGTTAAGTCACGATAATCAAACAGGAGAAACCATTATGATCGTTTCTGACATCGAAGCTAACGCCCTCTTAGAGAGCGTCACTAAGTTCCACTGCGGGGTTATCTACGACTACTCCACCGCTGAGTACGTAAGCTACCGTCCGAGTGACTTCGGTGCGTATCTGGATGCGCTGGAAGCCGAGGTTGCACGAGGCTGTCTTATTGTGTTCCACAACGGTCACAAGTATGACGTTCCTGCATTGACCAAACTGGCAAAGTtgcaattctgtc                                                     >  1:493181‑M2/10‑296 (MQ=255)`  
                                                                                                                                                                                                                                                           `tattgaCATTTGTTTCATATGAAGAGATTGTTAAGTCACGATAATC‑AATAGGAGAAATCAATATGATCGTTTCTGACATCGAAGCTAACGCCCTCTTAGAGAGCGTCACTAAGTTCCACTGCGGGGTTATCTACGACTACTCCACCGCTGAGTACGTAAGCTACCGTCCGAGTGACTTCGGTGCGTATCTGGATGCGCTGGAAGCCGAGGTTGCACGAGGCGGTCTTATTGTGTTCCACAACGGTCACAAGTATGACGTTCCTGCATTGACCAAACTGGCAAAGTTGCAATTGAACCgaga                                                 <  1:414260‑M2/295‑1 (MQ=255)`  
                                                                                                                                                                                                                                                            `tttgaCAGTTATTTCATAGGAAGAGATTGTAAAGTCAAGATAATC‑AATAGGAGAAATCAATGTGATCGTTTCTTACATGGAAGCTAACGCCCTCTTAGAGAGCGTCACTAAGTTCCACTGCGGGGTTATCTACGACTACTCCACCGCTGAGTACGTAAGCTACCGTCCGAGTGACTTCGGTGCGTATCTGGATGCGCTGGAAGCCGAGGTTGCACGAGGCGGTCTTATTGTGTTCCACAACGGTCACAAGTATGACGTTCCTGCATTGACCAAACTGGCAAAGTTGCAATTGAACCGagag                                                <  1:280403‑M2/296‑1 (MQ=255)`  
                                                                                                                                                                                                                                                                  `aGTTGTTTCATATGAAGAGATTGTTAAGTCACGATAATC‑AATAGGAGAAATCAATATGATCGTTTCTGACATCGAAGCTAACGCCCTCTTAGAGAGCGTCACTAAGTTCCACTGCGGGGTTATCTACGACTACTCCACCGCTGAGTACGTAAGCTACCGTCCGAGTGACTTCGGTGCGTATCTGGATGCGCTGGAATCCGAGGTTGCACGAGGCGGTCTTATTGTGTTCCACAACGGTCACAAGTATGACGTTCCTGCATTGACCAAACTGGCAAAGTTGCAATTGAACCGAGAGTTCCAc                                          <  1:263718/301‑1 (MQ=255)`  
                                                                                                                                                                                                                                                                          `tattagagacatttGTTAAGTCACGATAATC‑AATCGTAGAAATCAATCTGATCTTTTCTGACATCGAAGCTAACGCCCTCTTAGAGAGCGTCACTAAGTTCCACTGCGGGGTTATCTACGACTACTCCACCGCTGAGTACGTAAGCTACCGTCCGAGTGACTTCGGTGCGTATCTGGATGCGCTGGAAGCCGAGGTTGCACGAGGCGGTCTTATTGTGTTCCACAACGGTCACAAGTATGACGTTCCTGCATTGACCAAACTGGCAAAGTTGCAATTGAACCGAGAGTTCCACCTTCCTCg                                  <  1:46204/289‑1 (MQ=255)`  
                                                                                                                                                                                                                                                                          `cATATGAAGTGATTGTTAAGTCACGATAATC‑AATAGGAGAAATCAATATGATCGTTTCTGACATCGAAGCTAACGCCCTCTTAGAGAGCGTCACTAAGTTCCACTGCGGGGTTATCTACGACTACTCCACCGCTGAGTACGTAAGCTACCATCCGAGTGACTTCGGTGCGTATCTGGATGCGCTGGAAGCCGAGGTTGCACGAGGCGGTCTTATTGTGTTCCACAACGGTCACAAGTATGACGTTCCTGCATTGACCAAACTGGCAAAGTTGCAATTGAACCGAGAGTTCCACCTTCCTCg                                  <  1:403906/301‑1 (MQ=255)`  
                                                                                                                                                                                                                                                                            `tatGAAGAGATTGTTAAGTCACGATAATC‑AATAGGAGAAATCAATATGATCGTTTCTGACATCGAAGCTAACGCCCTCTTAGAGAGCGTCACTAAGTTCCACTGCGGGGTTATCTACGACTACTCCACCGCTGAGTACGTAAGCTACCGTCCGAGTGACTTCGGTGCGTATCTGGATGCGCTGGAAGCCGAGGTTGCACGAGGCGGTCTTATTGTGTTCCACAACGGTCACAAGTATGACGTTCCTGCATTGACCAAACTGGCAAAGTTGCAATTGAACCGAGAGTTCCACCTTCCTCGTg                                <  1:297945/301‑1 (MQ=255)`  
                                                                                                                                                                                                                                                                                 `agagATTGTTAAGTCACGATAATC‑AATAGGAGAAATCAATATGATCGTTTCTGACATCGAAGCTAACGCCCTCTTAGAGAGCGTCACTAAGTTCCACTGCGGGGTTATCTACGACTACTCCACCGCTGAGTACGTAAGCTACCGTCCGAGTGACTTCGGTGCGTATCTGGATGCGCTGGAAGCCGAGGTTGCACGAGGCGGTCTTATTGTGTTCCACAACGGTCACAAGTATGACGTTCCTGCATTGACCAAACTGGCAAAGTTGCAATTGAACCGAGAGTTCCACCTTCCTCGTGAGAAc                           <  1:73603/301‑1 (MQ=255)`  
                                                                                                                                                                                                                                                                                 `aaagaTTGTTAAGTCACGATAATC‑AATAGGAGAAATCAATATGATCGTTTCTGACATCGAAGCTAACGCCCTCTTAGAGAGCGTCACTAAGTTCCACTGCGGTGTTATCTACGACTACTCCACCGCTGAGTACGTAAGCTACCGTCCTAGTGACTTCGGTGCGTATCTGGATGCGCTGGAAGCCGAGGTTGCACGAGGCGGTCTTATTTTGTTCCACAACGGTCACAAGTATGACGTTCCTGCATTGACCAAACTGGCAAAGTTGCAATTGAACCTAGAGTTCCACCTTCCTCGTGAGAAc                           <  1:303747/299‑1 (MQ=255)`  
                                                                                                                                                                                                                                                                                        `gTTAAGTCACGATAATC‑AATAGGAGAAATCAATATGATCGTTTCTGACATCGAAGCTAACGCCCTCTTAGAGAGCGTCACTAAGTTCCACTGCGGGGTTATCTACGACTACTCCACCGCTGAGTACGTAAGCTACCGTCCGAGTGACTTCGGTGCGTATCTGGATGCGCTGGAAGCCGAGGTCGCACGAGGCGGTCCTATTGTGTTCCACAACGGTCACAAGTATGACGTTCCTGCATTGACCAAACTGGCAAAGTTGCAATTGAACCGAGAGTTCCCCCTTCCTCGTGAGCACTGTATTg                    >  1:274675/1‑301 (MQ=255)`  
                                                                                                                                                                                                                                                                                        `gTTAAGTCACGATAATC‑AATAGGAGAAATCAATATGATCGTTTCTGACATCGAAGCTAACGCCCTATTAGAGAGCGTCACTAAGTTCCACTGCGGGGTTATCTACGACTACTCCACCGCTGAGTACGTAAGCTACCGTCCGAGTGACTTCGGTGCGTATCTGGATGCGCTGGAAGCCGAGGTTGCACGAGGCGGTCTTATTGTGTTCCACAACGGTCACAAGTATGACGTTCCTGCATTGACAAAACTGGCAAAGTTGCAATTGAACCGAGAGTTCCACCTTCCTCGTGAGAACTGTAttt                    >  1:81766/1‑300 (MQ=255)`  
                                                                                                                                                                                                                                                                                          `tAAGTCACGATAATC‑AATAGGAGAAATCAATATGATCGTTTCTGACATAGAAGCTAACGCCCTCTGAGAGAGCGTCACTAAGTTCCACTGCGGGGTTATCTACGACTACTACACCGCTGAGTACGTAAGCTACCGTCCGAGTGACTTCGGTGCGTATCTGGATGCGCTGGAAGCCGAGGTTGCACGAGGCGGTCTTATTGTGTTCCACAACGGTCACAAGTATGACGTTCCTGCATTGACCAAACTGGCAAAGTTGCAATTGAACCGAGAGTTCCACCTTCCTCGTGAGAACTGTATTGac                  <  1:42173/301‑1 (MQ=255)`  
                                                                                                                                                                                                                                                                                          `aaaGTCACGATAATC‑AATAGGAGAAATAAATACGATCGTTTCTGACATCGAAGCTAACGCCCTCTTAGAGAGCGTCACTAAGTTCCACTGCGGGGTTATCTACGACTACTCCACCGCTGAGTACGTAAGCTACCGTCCGAGTGACTTCGGTGCGTATCTGGATGCGCTGGAAGCCGAGGTTGCACGAGGCGGTCTTATTGTGTTCCACAACGGTCACAAGTATGACGTTCCTGCATTGACCAAACTGGCAAAGTTGCAATTGAACCTAGAGTTCCACCTTCCTCGTGAGAACTGTATTGac                  <  1:487262/300‑1 (MQ=255)`  
                                                                                                                                                                                                                                                                                           `caGTCACGATAATC‑AATAGGAGAAATCAATATGATCGTTTCTGACATCGAAGCTAACGCCCTCTTAGAGAGCGTCACTAAGTTCCACTGCGGGGTTATCTACGACTACTCCACCGCTGAGTACGTAAGCTACCGTCCGAGTGACTTCGGTGCGTATCTGGATGCGTTGGAAGCCGAGGTTGCACGAGGCGGTCTTATTGTGTTCCACAACGGTCACAAGTATGACGTTCCTGCATTGACCAAACTGGCAAAGTTGCAATTGAACCGAGAGTTCAACCTTCCTCGTGAGAACGGTATTGaca                 >  1:258623/2‑301 (MQ=255)`  
                                                                                                                                                                                                                                                                                            `aGTCACGATAATC‑AATAGGAGAAATCAATATGATCGTTTCTGACATCGAAGCTAACGCCCTCTTAGAGAGCGTCACTAAGTTCCACTGCGGGGTTATCTACGACTACTCCACCGCTGAGTACGTAAGCTACCGTCCGAGTGACTTCGGTGCGTATCTGGATTCGCTGTAAGCCGAGGTTGCACGAGGCGGTCTTATTGTGTTCCACAACGGTCACAAGTATGACGTTCCTGCATTGACCAAACTGGCAAAGTTGCAATTGAACCGAGAGTTCCACCTTCCTCGTGAGAACTGTATTGacac                <  1:381434/301‑1 (MQ=255)`  
                                                                                                                                                                                                                                                                                            `aGTCACGATAATC‑AATAGAAGAAATCAATATGATCGTTTCTGGCATCGAAGCTAACGCCCTCTTAGAGAGCGTCACTAAGTTCCACTGCGGGGTTATCTACGACTACTCCACCGCTGAGTACGTAAGCTACCGTCCGAGTGACTTCGGTGCGTATCTGGATGCGCTGGAAGCCGAGGTTGCACGAGGCGGTCTTATTGTGTTCCACAACGGTCACAAGTATGACGTTCCTGCATTGACCAAACTGGCAAAGTTGCAATTGAACCGAGAGTTCCACCTTCCTCGTGAGAACTGTATTGacac                <  1:328437/301‑1 (MQ=255)`  
                                                                                                                                                                                                                                                                                             `gtaaCGATAATC‑AATAGGAGAAATCAATATGATCGTTTCTGACATCGAAGCTAACGCCCTCTTAGAGAGCGTCACTAAGTTCCACTGCGGGGTTATCTACGACTACTCCACAGCTGAGTACGTAAGCTACCGTCCGAGTGACTTCGGTGCGTATCTGGATGCGCTGGAAGCCGAGGTTGCACGAGGCGGTCTTATTGTGTTCCACAACAGTCACAAGTATGACGTTCCTGCATTGACCAAACTGGCAAAGTTGCAATTGAACCGAGAGTTCCACCTTCCTCGTGAGAACTGTATTGACAcc               <  1:130023/298‑1 (MQ=255)`  
                                                                                                                                                                                                                                                                                                 `ggATAATC‑AATAGGGGAAATCAAGATGATCGTTTCGGACCTCGAAGCTAACGCCCTTTTAGAGAGCGTCACTAAGTTCCACAGCGGGGTTATCTACGACTACTCCACCGCTGAGTACGTAAGCTACCGTCCGAGTAACTTCGGTGCGTATCTGGATGCGCTGGAAGCCGAGGTTGCACGAGGCGGTCTTATTGTGTTCCACAACGGTAACAAGTATGACGTTCCTGCATTGACCAAACTGGCAAAGGTGCAATTGAACCGAGAGTTCCACCTTCCTCGTGAGAACTGTATTGATACCCTtg           <  1:509848/300‑1 (MQ=255)`  
                                                                                                                                                                                                                                                                                                 `cGATAATC‑AATAGGATAAATCAATATGATCGTTTCTGACATCGAAGCTAACGCCCTCTTAGAGAGCGTCACTAAGTTCCACTGCGGGGTTATCTACGACTACTCCACCGCTGAGTACGTAAGCTACCGTCCGAGTGACTTCGGTGCGTATCTGGATGCGCTGGAAGCCGAGGTTGCACGAGGCGGTCTTATTGTGTTCCACAACGGTCACAAGTATGACGTCCCTGCATTGACCAAACTGGCAAAGTTGCAATTGAACCGAGAGTTCCACCTTCCTCGTGAGAACTGTATTGACACCAttt           >  1:315369/1‑300 (MQ=255)`  
                                                                                                                                                                                                                                                                                                 `cGATAATC‑AATAGGAGAAATCAATATGATCGTTTCTGACATCGAAGCTAACGCCCTCTTAGAGAGCGTCACTTTTTTCCACTGCGTTTTTCTCTACGACTTCTCCTCCGCTGATTTCTTTAGCTACCTTCCTCTTGACTTCTTTTCTTATCTGGATGCGCTGGAAGCCGTGGTTTCACGAGTCGGTCTTTTTGTGTTCCACATCGTTCACAAGTATGACGTTCCTTCTTTTACCAAACTGTCAATGTTTCTATTTAACCGAGAGTTCCACCTTCCTCGTGAGAACttttttttcttttttt           >  1:126030/1‑286 (MQ=255)`  
                                                                                                                                                                                                                                                                                                 `cGATAATC‑AATAGGAGAAATCAATATGATCGTTTCTGACATCGAAGCTAACGCCCTCTTAGAGAGCGTCACTAAGTTCCACTGCGGGGTTATCTACGACTACTCCACCGCTGAGTACGTAAGCTACCGTCCGAGTGACTTCGGTGCGTATCTGGATGCGCTGGAAGCCGAGGTTGCACGAGGCGGTCTTATTGTGTTCCACAACTGTCACAAGTATGACGTTCCTGCATTGACCAAACTGGCAAAGTTGCAATTGAACCGAGAGTTCCACCTTCCTCGTGAGAACTGTATTGACACCCTtg           <  1:323351/301‑1 (MQ=255)`  
                                                                                                                                                                                                                                                                                                   `aTAATC‑AATAGGAGAAACCATTATGATCGTTTCTGACATCGAAGCTAACGCCCTCTTAGAGAGCGTCACTAAGTTCCACTGCGGGGTTATCTACGACTACTCCACCGCTGAGTACGTAAGCTACCGTCCGAGTGACTTCGGTGCGTATCTGGATGCGCTGGAAGCCGAGGTTGCACGAGGCGGTCTTATTGTGTTCCACAACGGTCACAAGTATGACGTTCCTGCATTGACCAAACTGGCAACATTGCAATTGAACCGAGAGTTCCACCTTCCTCTTGAGAACTGTATTGCCAcctgtctc         >  1:345366/1‑295 (MQ=255)`  
                                                                                                                                                                                                                                                                                                       `gtgtaTAAGAGACAGCAATATGATCGTTTCTGACATCGAAGCTAACGCCCTCTTAGAGAGCGTCACTAAGTTCCACTGCGGGGTTATCTACGACTACTCCACCGCTGAGTACGTAAGCTACCGTCCGAGTGACTTCGGTGCGTATCTGGATGCGCTGGAAGCCGAGGTTGCACGAGGCGGTCTTATTGTGTTCCACAACGGTCTCAAGTATGACGTTCCTGCATTGACCAAACTGGCAAAGTTGCAATTGAACCGAGAGTTCCACCTTCCTCGTGAGAACTGTATTGACACCCTTGtgttg      <  1:336300/297‑1 (MQ=255)`  
                                                                                                                                                                                                                                                                                                          `aaTAGGAGAAATCAATATGATCGTTTCTGACATCGAAGCTAACGCCCTCTTAGAGAGCGTCACTAAGTTCCACTGCGGGGTTATCTACGACTACTCCACCGCTGAGTACGTAAGCTACCGTCCGAGTGACTTCGGTGCGTATCTGGATGCGCTGGAAGCCGAGGTTGCACGAGGCGGTCTTATTGTGTTCCACAACGGTCACAAGTATGACGTTCCTGCATTGACCAAACTGGCAAAGTTGCAATTGAACCGAGAGTTCCACCTTCCTCGTGAGAACTGTATTGACACCCTTGTGTTGTCa   <  1:94760/301‑1 (MQ=255)`  

                                                                                                                                                                                                                                                                                                            `|`                                                                                                                                                                                                                                                                                                             
`AGGCTGGACTTGGACAAGACAGACTGGCTGGAAGAAACCAGAAGTTACCAAGCTGAGTCATAAGGTGTTCGATAAGGACACTATGACCCACATCAAGGCTGGTGATTGGGTTAAGGTTGACATGGGAGTTGTTGGTGGATACGGCTACGTCCGCTCAGTTAGTGGCAAATATGCACAAGTGTCATACATCACAGGTGTTACTCCACGCGGTGCAATCGTTGCCGATAAGACCAACATGATTCACACAGGTTTCTTGACAGTTGTTTCATATGAAGAGATTGTTAAGTCACGATAATC‑AATAGGAGAAATCAATATGATCGTTTCTGACATCGAAGCTAACGCCCTCTTAGAGAGCGTCACTAAGTTCCACTGCGGGGTTATCTACGACTACTCCACCGCTGAGTACGTAAGCTACCGTCCGAGTGACTTCGGTGCGTATCTGGATGCGCTGGAAGCCGAGGTTGCACGAGGCGGTCTTATTGTGTTCCACAACGGTCACAAGTATGACGTTCCTGCATTGACCAAACTGGCAAAGTTGCAATTGAACCGAGAGTTCCACCTTCCTCGTGAGAACTGTATTGACACCCTTGTGTTGTCAC  >  NC_001604.1/14040‑14638`

  

Alignment Legend

Aligned base mismatch/match (shaded by quality score): `ATCG/ATCG` `< 3 ≤` `ATCG/ATCG` `< 10 ≤` `ATCG/ATCG` `< 21 ≤` `ATCG/ATCG` `< 34 ≤` `ATCG/ATCG` `< 38 ≤` `ATCG/ATCG`

Unaligned base: `atcg`    Masked matching base: `atcg`    Alignment gap: `‑`    Deleted base: `‑`

Reads not counted as support for junction

read\_name Not counted due to insufficient overlap past the breakpoint.

read\_name Not counted due to not crossing MOB target site duplication.
